# Supplementary material for: Non-rhythmic head-direction cells in the parahippocampal region are not constrained by attractor network dynamics
Source: eLife. 2018 Sep 17;7:e35949. doi: 10.7554/eLife.35949 (PMC6158010; doi:10.7554/eLife.35949)
Supplement: Supplementary file 1. — Location of the tetrode tips and number of HD cells (theta-rhythmic and non-rhythmic) and grid cells recorded in each hemisphere. [file elife-35949-supp1.pdf]

		Tetrode tips							Cells				
**Mouse**	**Hemisphere**	**MEC**	**PaS/ MEC**	**PaS**	**PrS/ PaS**	**PrS**	**PrS/ RSA**	**Cortex**	**HD cells**	**Theta-rhythmic**	**Non-rhythmic**	**Grid cells**	**All cells**
jp757	left	1	0	0	2	0	0	0	11	4	7	9	65
jp757	right	1	0	0	0	0	0	0	8	1	7	19	61
jp759	left	4	0	0	0	0	0	0	0	0	0	13	27
jp759	right	2	0	0	0	0	0	0	10	3	7	31	92
jp865	left	0	0	0	0	2	1	0	0	0	0	11	46
jp865	right	3	0	0	0	0	0	1	7	7	0	2	39
jp2097	left	0	0	4	0	0	0	0	2	2	0	1	30
jp2097	right	2	0	0	0	0	0	0	17	16	1	8	64
jp2158	left	0	0	2	0	0	0	0	0	0	0	0	47
jp2158	right	4	0	0	0	0	0	0	2	2	0	23	93
jp2159	left	3	0	0	0	0	0	0	4	3	1	30	113
jp2159	right	2	0	0	0	0	0	0	8	2	6	33	84
jp3302	left	0	0	1	0	0	2	0	0	0	0	0	3
jp3302	right	2	0	0	0	0	0	0	8	7	1	0	22
jp4506	left	2	2	0	0	0	0	0	6	5	1	20	82
jp4506	right	4	0	0	0	0	0	0	9	7	2	11	52
jp4688	left	0	0	2	1	0	0	0	0	0	0	7	10
jp4688	right	4	0	0	0	0	0	0	1	0	1	1	14
**Total**		**34**	**2**	**9**	**3**	**2**	**3**	**1**	**93**	**59**	**34**	**219**	**944**
