# Supplementary material for: Non-rhythmic head-direction cells in the parahippocampal region are not constrained by attractor network dynamics
Source: eLife. 2018 Sep 17;7:e35949. doi: 10.7554/eLife.35949 (PMC6158010; doi:10.7554/eLife.35949)
Supplement: Transparent reporting form [file elife-35949-transrepform.pdf]

eLIFE
1st Floor
24 Hills Road
Cambridge CB2 1JP, UK
P 01223 855340
W elifesciences.org
T @elife

eLife statistical submission form

We encourage authors to provide detailed information *within their submission* to facilitate the interpretation and replication of experiments. If you have any questions, please contact us: editorial@elifesciences.org.

Sample-size estimation

- You should state whether an appropriate sample size was computed when the study was being designed
- You should state the statistical method of sample size computation and any required assumptions
- If no explicit power analysis was used, you should describe how you decided what sample (replicate) size (number) to use

Please outline where this information can be found within the submission (e.g., page numbers or figure legends), or explain why this information doesn't apply to your submission:

No power analysis was used. We decided to have 9 mice for the experiment to ensure that the effects reported in the manuscript were present in most animals. We recorded MEC neurons from 9 mice. 944 neurons were analyzed. The statistical units throughout the manuscript were neurons. For most statistical comparisons, we used non-parametric test including bootstrap tests to increase power.

None of the major conclusions of the manuscript was based on negative results (failing to find a statistical difference).

Information regarding sample size used for specific tests is reported throughout the results section.

Replicates

- You should report how often each experiment was performed
- You should include a definition of biological versus technical replication
- The data obtained should be provided and sufficient information should be provided to indicate the number of independent biological and/or technical replicates
- If you encountered any outliers, you should describe how these were handled
- Criteria for exclusion/inclusion of data should be clearly stated

eLife Sciences Publications, Ltd is a limited liability non-profit non-stock corporation incorporated in the State of Delaware, USA, with company number 5030732, and is registered in the UK with company number FC030576 and branch number BR015634 at the address 1st Floor, 24 Hills Road, Cambridge CB2 1JP | August 2014

- High-throughput sequence data should be uploaded before submission, with a private link for reviewers provided (these are available from both GEO and ArrayExpress)

Please outline where this information can be found within the submission (e.g., page numbers or figure legends), or explain why this information doesn't apply to your submission:

We recorded MEC neurons from 9 mice.

No data points were excluded from the analysis.

This information can be found in the Results section (line 100).

Statistical reporting

- Statistical analysis methods should be described and justified
- Raw data should be presented in figures whenever informative to do so (typically when N per group is less than 10)
- For each experiment, you should identify the statistical tests used, exact values of N, definitions of center, methods of multiple test correction, and dispersion and precision measures (e.g., mean, median, SD, SEM, confidence intervals; and, for the major substantive results, a measure of effect size (e.g., Pearson's r, Cohen's d)
- Report exact p-values wherever possible alongside the summary statistics and 95% confidence intervals. These should be reported for all key questions and not only when the p-value is less than 0.05.

Please outline where this information can be found within the submission (e.g., page numbers or figure legends), or explain why this information doesn't apply to your submission:

eLife Sciences Publications, Ltd is a limited liability non-profit non-stock corporation incorporated in the State of Delaware, USA, with company number 5030732, and is registered in the UK with company number FC030576 and branch number BR015634 at the address 1st Floor, 24 Hills Road, Cambridge CB2 1JP | August 2014

For most statistical analysis, we used non-parametric tests that do not rely on assumptions about the shape of the distributions.

When statistical tests were reported, we gave the name of the test, the value of the test statistic and the exact values of *n* and *p*.

For important tests, we showed the distribution of the raw data points (Figures 4c-d, 5b, 6e, 7b, 8e), a boxplot (median, 1st and 3rd quartiles; Figures Figure 1-figure supplement 2f-g, Figure 3c, Figure 3f, Figure 4c-d, Figure5b, Figure6e) or the raw data points (Figure 1f).

The statistical tests used were reported on lines 134, 160, 162, 164, 173, 176, 179, 191, 193, 208, 210, 236, 238, 254, 258, 270, 279, 324, 326, 349, 351, 357, 366, 369, 381, 383, 400, 413, 416.

(For large datasets, or papers with a very large number of statistical tests, you may upload a single table file with tests, Ns, etc., with reference to page numbers in the manuscript.)

Additional data files ("source data")

- We encourage you to upload relevant additional data files, such as numerical data that are represented as a graph in a figure, or as a summary table
- Where provided, these should be in the most useful format, and they can be uploaded as "Source data" files linked to a main figure or table
- Include model definition files including the full list of parameters used
- Include code used for data analysis (e.g., R, MatLab)
- Avoid stating that data files are "available upon request"

Please indicate the figures or tables for which source data files have been provided:

The electrophysiological data have been uploaded to the Dryad database. The source code for the analysis is on GitHub.

eLife Sciences Publications, Ltd is a limited liability non-profit non-stock corporation incorporated in the State of Delaware, USA, with company number 5030732, and is registered in the UK with company number FC030576 and branch number BR015634 at the address 1st Floor, 24 Hills Road, Cambridge CB2 1JP | August 2014
